# Unusual Presentation of Multiple Lung Nodules in a Patient With Supraglottic Squamous Cancer: A Rare Infectious Cause Revealed

**DOI:** 10.7759/cureus.43796

**Published:** 2023-08-20

**Authors:** Zakaria Alagha, Ibrahim Shanti, Muhammad Ghallab, Amro Al-Astal

**Affiliations:** 1 Internal Medicine, Marshall University Joan C. Edwards School of Medicine, Huntington, USA; 2 Internal Medicine, Icahn School of Medicine at Mount Sinai, NYC Health and Hospitals, Queens, New York, USA; 3 Internal Medicine/Pulmonology, Marshall University Joan C. Edwards School of Medicine, Huntington, USA

**Keywords:** pulmonary critical care, bronchoscopy, corynebacterium spp, laryngeal tumors, multiple nodules in the lung

## Abstract

Pulmonary nodules are commonly encountered in medical practice, necessitating thorough evaluation due to their diverse etiologies. Identifying the underlying cause is of utmost importance, particularly in patients with a history of extrapulmonary cancer, to differentiate between metastasis and other etiologies. We present a rare case of a 24-year-old male with supraglottic squamous cancer who developed multiple pulmonary nodules, which surprisingly were caused by a rare infectious agent. The patient presented with bilateral infiltrates on imaging, raising strong suspicion of metastatic disease from primary cancer. However, bronchoscopy and biopsy revealed no malignancy but confirmed the presence of *Corynebacterium amycolatum*, leading to a change in the treatment approach from palliative to curative. This case highlights the importance of considering other etiologies, especially infections, in patients with cancer and pulmonary nodules. Accurate diagnosis is crucial to guide appropriate management decisions and optimize patient outcomes.

## Introduction

Pulmonary nodules, characterized as small (< 3 cm) lesions in the lung parenchyma, are a common radiological finding that can have diverse clinical implications [1]. Their presence can raise concerns about underlying conditions such as infections, inflammation, or even metastatic malignancies. While metastasis is a well-known cause of multiple lung nodules, other rare etiologies need to be considered, especially when they coexist with primary cancer [2]. This case report highlights the need to recognize and investigate uncommon presentations of pulmonary nodules in patients with cancer. We present a unique case of a patient with supraglottic squamous cancer who developed multiple pulmonary nodules, and unexpectedly, these nodules were not indicative of metastasis. Instead, a rare infectious agent, *Corynebacterium amycolatum*, was identified as the underlying cause, shedding light on the need for a comprehensive evaluation in such scenarios.

## Case presentation

**Figure 1:**
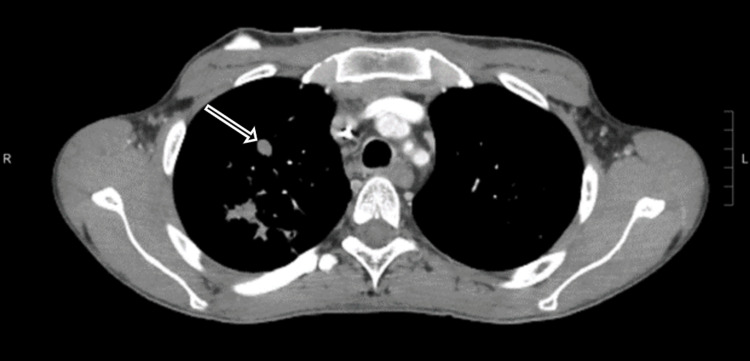
Soft-tissue window 1-mm transverse CT sections show a smoothly marginated 1 cm solid nodule (arrow) in the upper lobe of the right lung

A 24-year-old white male with a medical history of supraglottic squamous cancer underwent concurrent chemoradiation with ongoing cisplatin therapy. The patient had a Medi-port and tracheostomy tube in situ. He presented with a productive cough with yellow sputum and facial swelling. Upon admission, vital signs were significant for a temperature of 99.7 Fahrenheit, heart rate of 99 beats per minute, blood pressure of 106/62 mmHg, and respiratory rate of 14 breaths per minute. Physical examination revealed decreased bilateral breath sounds; otherwise, it was unremarkable. Laboratory investigations revealed a significantly elevated erythrocyte sedimentation rate (ESR) of 86 mm/hr (normal range: 0-15 mm/hr in men) and a C-reactive protein (CRP) level of 13.3 mg/dl (normal range: less than 0.3 mg/dL). An initial chest X-ray demonstrated bilateral patchy infiltrates. Subsequent contrast-enhanced CT scan of the chest disclosed multiple pulmonary nodules measuring 1-5 cm, along with new patchy consolidation in the right upper lobe and bilateral ground-glass opacities, prompting strong suspicion of metastasis from primary cancer, in addition to possible bilateral pneumonia (Figure 1). To definitively rule out infective endocarditis, transthoracic echocardiography was performed, yielding negative results. Despite initial concerns for Lemmierre disease due to facial edema, the venous duplex of jugular veins effectively ruled out thrombosis.

Bronchoscopy was performed for further evaluation of the lung nodules. Analysis of bronchoalveolar lavage exhibited a significant presence of neutrophils while acid-fast bacilli testing returned negative results, and no microorganisms were detected on Gram stain. Histopathological examination of the bronchoalveolar specimen from the right upper lobe posterior segment demonstrated benign bronchial mucosa and lung parenchyma, along with nonspecific chronic inflammation of alveolar walls and an increased number of pigment-laden macrophages. Malignancy was not identified in the tissue biopsy; however, the intriguing finding was the positive results obtained from the tissue culture with gram stain, indicating the presence of *Corynebacterium amycolatum*. The patient's treatment initially involved the administration of vancomycin and ampicillin-sulbactam, which was later modified to amoxicillin-clavulanic acid and linezolid upon discharge, with a total antibiotic course lasting for three weeks. Subsequently, the patient exhibited clinical improvement, with ESR and CRP levels returning to within normal ranges.

## Discussion

Pulmonary nodules are frequently detected incidentally when a chest X-ray or a CT scan is conducted for unrelated reasons [1]. They are identified in approximately 1.6 million people per year in the United States, constituting approximately 30% of chest CT scans [3]. Pulmonary nodules can have various underlying causes such as primary lung neoplasms, metastatic nodules, infectious diseases, and others. Numerous studies have reported a notable incidence of secondary malignancies in patients with extrapulmonary cancers, underscoring the significance of accurately identifying the precise nature of these nodules [4]. For individuals who have a past history of extrapulmonary cancer, it is of paramount importance to refrain from automatically assuming that lung nodules are metastatic in nature. Erroneous assumptions can lead to the initiation of a different therapeutic approach, involving chemotherapy, radiation, or even ablative therapy. Such misinterpretations may impact the cancer staging, changing it from localized cancer with a probable favorable prognosis to metastatic cancer, necessitating a palliative treatment approach. Careful and accurate assessment of these nodules is paramount to ensure appropriate and tailored management strategies [5]. Radiological diagnosis serves as the primary step in identifying these nodules. For instance, a study was conducted to assess the significance of pulmonary nodules detected on thin-section CT chest in patients with extrapulmonary malignant neoplasms. Their findings indicated that most of these nodules, particularly those larger than 10 mm in size or located more than 10 mm away from the pleura, were associated with malignancy [2]. Additionally, Cahan WG and colleagues conducted a study involving 800 patients with a history of known cancer who were presented with solitary pulmonary nodules. These patients underwent thoracotomy to determine the nature of these nodules. The study revealed that most nodules were either primary lung cancer or metastasis, with only 11 cases identified as benign. This emphasizes the importance of accurate diagnosis and the potential implications for treatment decisions in patients with pulmonary nodules and a history of extrapulmonary cancer [5]. A separate study involving 151 patients with extrapulmonary cancer and non-calcific pulmonary nodules showed that approximately half of these nodules were malignant. This finding further emphasizes the significance of meticulous evaluation and precise diagnosis in patients with extrapulmonary cancer, as a considerable proportion of these nodules may indicate malignancy and require appropriate management strategies [6].

In the case of patients diagnosed with head and neck cancer, the rate of isolated pulmonary metastasis is estimated to be around 20-30%. This highlights the need for a vigilant assessment of pulmonary nodules in this patient population to distinguish between metastatic lesions and other potential causes [7]. Bronchoscopy with subsequent histological diagnosis is essential for the accurate evaluation of pulmonary nodules. It exhibits a high sensitivity in detecting a malignant process in solitary lung nodules, ranging from approximately 20% to 80%, depending on the size and location of the nodule. The sensitivity can reach up to 70% when the bronchus is leading to the lesion. One study evaluated the diagnostic yield of bronchoscopy in 113 patients with proven metastasis who have CT findings of solitary or multiple pulmonary nodules. The highest diagnostic yield was for metastasis from head and neck cancer [8,9].

Non-diphtheria *Corynebacterium* species are characterized as facultative, intracellular, non-spore-forming, irregularly shaped gram-positive bacilli. Although these species normally reside as commensal flora in the respiratory tract, skin, and mucous membranes, they are increasingly being recognized as emerging pathogens with only a limited number of reported cases of respiratory tract infections. Pathogenicity is established when an abundant number of *Corynebacterium *is observed in microscopic fields containing more than 20 white blood cells, particularly when the bacteria are found within polymorphonuclear cells (PMNs) and are supported by a positive culture result. *Corynebacterium *pneumonia is predominantly reported in patients with bypassed upper respiratory tracts, such as those with tracheostomies, or in individuals who are immunocompromised, as exemplified by our patient undergoing cisplatin therapy. While presenting with similarities to other types of bacterial pneumonia, the utility of blood cultures in detecting these species is minimal. In terms of management, all *Corynebacterium *species are susceptible to vancomycin, linezolid, and tigecycline, with Vancomycin being the recommended initial treatment choice. Prompt recognition and appropriate management of *Corynebacterium *pneumonia are crucial to ensure effective patient care and favorable outcomes [10,11].

This case report highlights the challenges in accurately diagnosing pulmonary nodules in patients with extrapulmonary malignancies, emphasizing the need to consider infectious causes as part of the differential diagnosis. The findings underscore diagnostic challenges in distinguishing between metastatic nodules and other pathologies. Future research may focus on developing diagnostic algorithms for the diagnosis of pulmonary nodules in patients with extrapulmonary malignancy.

## Conclusions

This case underscores the significance of a meticulous approach to managing multinodular pulmonary disease in patients with a current or prior cancer diagnosis. Our unexpected findings challenge conventional assumptions revealing an infectious cause instead of metastasis. Precise diagnosis can reshape treatment strategies from palliative to curative intent, emphasizing the importance of cautious and innovative patient-centered care.
